# Early detection of paediatric and adolescent obsessive–compulsive, separation anxiety and attention deficit hyperactivity disorder using machine learning algorithms

**DOI:** 10.1007/s13755-023-00232-z

**Published:** 2023-07-22

**Authors:** Umme Marzia Haque, Enamul Kabir, Rasheda Khanam

**Affiliations:** 1 School of Mathematics, Physics and Computing, University of Southern Queensland, Toowoomba, Australia; 2 School of Business, University of Southern Queensland, Toowoomba, Australia

**Keywords:** OCD, ADHD, SAD, CV, ML

## Abstract

**Purpose:**

Mental health issues of young minds are at the threshold of all development and possibilities. Obsessive–compulsive disorder (OCD), separation anxiety disorder (SAD), and attention deficit hyperactivity disorder (ADHD) are three of the most common mental illness affecting children and adolescents. Several studies have been conducted on approaches for recognising OCD, SAD and ADHD, but their accuracy is inadequate due to limited features and participants. Therefore, the purpose of this study is to investigate the approach using machine learning (ML) algorithms with 1474 features from Australia's nationally representative mental health survey of children and adolescents.

**Methods:**

Based on the internal cross-validation (CV) score of the Tree-based Pipeline Optimization Tool (TPOTClassifier), the dataset has been examined using three of the most optimal algorithms, including Random Forest (RF), Decision Tree (DT), and Gaussian Naïve Bayes (GaussianNB).

**Results:**

GaussianNB performs well in classifying OCD with 91% accuracy, 76% precision, and 96% specificity as well as in detecting SAD with 79% accuracy, 62% precision, 91% specificity. RF outperformed all other methods in identifying ADHD with 91% accuracy, 94% precision, and 99% specificity.

**Conclusion:**

Using Streamlit and Python a web application was developed based on the findings of the analysis. The application will assist parents/guardians and school officials in detecting mental illnesses early in their children and adolescents using signs and symptoms to start the treatment at the earliest convenience.

## Introduction

Mental health, often known as social and emotional well-being, is essential for healthy child development. Among the most common mental illnesses affecting children and adolescents are obsessive–compulsive disorder (OCD), separation anxiety disorder (SAD), and attention deficit hyperactivity disorder (ADHD). According to the most recent statistics of mental disorders in Australia, around 1 in every 7 children and adolescents aged 4–17 years has a mental disorder, which equals to approximately 560,000 Australian children and adolescents [1]. One of these, OCD, which is one of the 10 leading causes of years spent disabled over the world, that affects about half a million Australians or 2–3% of the population [2]. Also worth mentioning is SAD, which affects about 4% of Australian children whose ages are between 4 and 17 years, that have a significant negative impact on their life [3]. A very worrisome fact is that ADHD affects children (5–10%) more severely than adults (4%) [4]. It is currently the most common paediatric diagnosis in Australia, and general paediatricians, who serve as primary healthcare providers, diagnose 18% of ADHD patients who attend for care [5].

Machine learning (ML) has emerged as a powerful tool for making critical decisions through the analysis of large datasets, such as social behaviour patterns and various health conditions [6–9]. Although several studies have been conducted on obsessive–compulsive disorder (OCD) and attention-deficit/hyperactivity disorder (ADHD), this study focuses on reviewing the existing literature on ML studies conducted on children and youth with emphasis on the most cited studies with high accuracy, area under receiver operating characteristic curve (AUC) score, sensitivity, and specificity. However, there is a dearth of research on the use of ML algorithms for detecting separation anxiety disorder (SAD). Summaries of the methods used in a previous synthesis of related literature and their results are shown in Table 1.

**Table 1:** Summaries of the methods and outcomes of previous literature reviews

Method/classifier	Dataset	Performance metric	References
ADHD detection			
Support vector Machine	50 individuals of 10–12 aged school children in Chile with their oculometry data (such as, gaze position, blink frequency and pupil size) from 22 custom engineered features	75% accuracy, 77% sensitivity and 74% specificity	[10]
Linear discriminant analysis	5726 college students	93.1% accuracy	[11]
Support Vector Machine	150 individuals from the national institutes in Boston, USA	96.5% accuracy	[12]
Support Vector Machine	36 ADHD and 35 normal cases of a private dataset	74.65% accuracy, 75% sensitivity and 74.29% specificity	[13]
Single-channel deep neural network	Multiscale brain connectome data and personal characteristic data of 973 participants from Neuro Bureau ADHD-200 dataset	82% AUC	[14]
Support Vector Machine with Recursive Feature Elimination	159 structural MRI images of children from Neuro Bureau ADHD-200 dataset	60.78% accuracy	[15]
Extreme learning machine	776 individuals from 7 to 14 years with their structural MRI data from Neuro Bureau ADHD-200 dataset	76.19% accuracy	[16]
Support Vector Machine	973 participants including ADHD patients and healthy controls from Neuro Bureau ADHD-200 dataset	75% accuracy	[17]
Boruta based feature selection and support vector machine for identification	Comorbidity-free ADHD individuals with covariable-matched healthy children aged 9–10 chosen from the Adolescent Brain and Cognitive Development study	64.3% accuracy and 69.8% AUC	[18]
Support vector machine	fMRI scan and phenotypic data (age, gender, handedness, IQ, and site of scanning) of 668 participants from the ADHD-200 dataset	76% accuracy on 2 class and 68.6% on 3 class accuracy	[19]
Novel Knowledge Distillation-Based Feature Selection based on neural network	776 training and 197 testing cases from the ADHD-200 dataset	Average 75% accuracy on different sites	[20]
Linear support vector machine	ADHD-200 data set rom Kennedy Krieger Institute (KKI), NeuroImage (NI), New York University Medical Center (NYU) and Peking University (Peking)	56–81% accuracy on different dataset	[21]
Support vector machine-recursive feature elimination	83 boys aged 7–15 with ADHD, 86 boys with ASD, 125 boys with typical development	79.3% accuracy	[22]
OCD detection			
Logistic regression in neural network	47 children and adolescents with OCD and 17 children and adolescents without a psychiatric diagnosis, aged 8–17 years	89% accuracy, 78% sensitivity, 86% specificity, and an 89% AUC score	[23]
Gradient enhanced decision trees	215 observations and 227 features	AUROC score of 78.2%, sensitivity of 73.42%, and specificity of 71.45%	[24]
Random Forest	533 participants of a specialised outpatient clinic	65% accuracy	[25]
Random Forest	61 adolescents with 46 demographic and clinical baseline variables by Child and Adolescent Psychiatry Research Centre in Stockholm, Sweden	83% accuracy	[26]
Support Vector Machine	68 drug naïve OCD patients of Chinese Han nationality	72% accuracy	[27]
Ensemble method	330 Iranian patients with 36 features	86% accuracy	[28]
Support Vector Machine	296 individuals using 24 baseline variables	75.4% accuracy	[29]
Support Vector Machine	54 drug naïve Chinese OCD patients with 6 motion parameters	Accuracy 95.37%, sensitivity 96.30%, and specificity 94.44%,	[30]
SAD detection			
Alternating decision trees	Children aged 2–5 who visited Duke University Paediatric Primary Care Clinics	96% accuracy	[31]

As can be seen in Table 1, ML algorithms have previously been applied to the detection of OCD and ADHD, but there has been little research on using ML algorithms to detect SAD. These prior research results demonstrate good value with accuracy, although they addressed a small number of participants and had inter-participant heterogeneity due to data obtained from several sites [10–31]. Most literature has used ML algorithms with MRI images, EEG, and ECG data to create an automated diagnosis tool so a diagnosis expert can quickly recognise this without clinical tests. Only clinical specialists can use these tools because the general public cannot access this input information.

There is increasing evidence that Australian children and adolescents with certain mental illnesses go untreated because their family, caretakers, teachers, or school officials are unaware of the symptoms. Thus, many children who could benefit from early diagnosis go untreated, affecting their education, social and emotional development, and future employment. To the best of our knowledge, no study has identified these mental health issues in Australian children and adolescents using ML algorithms. This study, therefore, aimed to close the gap to identify the determinants of these mental disorders so that the non-clinical persons such as parents, teachers, and school officials between children and adolescents in the Australian context can make an informed decision to initiate treatment without any further delay. Several algorithms have been put into place after thoroughly considering the options for ML techniques in order to pinpoint characteristics most closely connected with OCD, SAD, and ADHD, in particular:

- 1. Design and validate a new framework to present the actual signs or symptoms of ADHD, OCD and SAD that children and adolescents in Australia are encountering.
- 2. Evaluate the supervised models’ performances in terms of measuring accuracy, sensitivity, specificity, precision, AUC score and K-Fold cross validation score.
- 3. Show how the best fitted model for OCD, SAD, and ADHD prediction is employed to generate detection decisions using the most important input features for sample test cases.
- 4. Develop a web application in order to use this model as a viable tool to detect these mental illnesses at their early stage.

The remaining paper is structured as follows: in “Materials and methods”section provides a detailed overview of the materials and methods that have been adopted. This section briefly discusses the dataset, the features extraction methods and the ML algorithms used in this paper. In “Results and discussions” section shows the experimental results that have been achieved after applying the classification algorithms with a detailed discussion. Finally, “Conclusion” section provides conclusions and summarises the study.

## Materials and methods

**Fig. 1:**
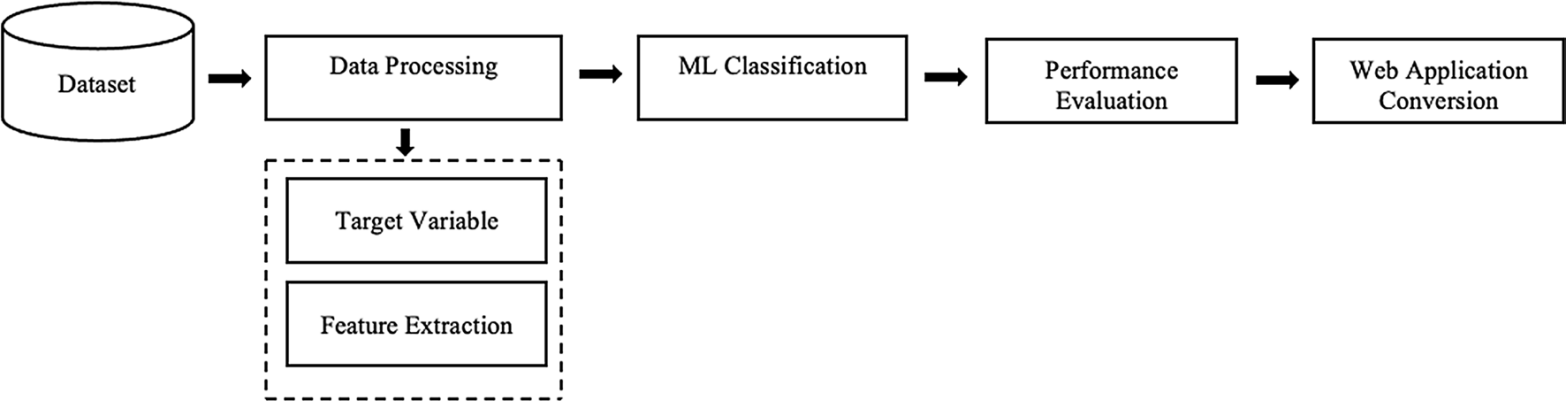
ML based predictive model detection process

This section presents the proposed framework for the classification tasks of OCD, SAD, and ADHD by using ML algorithms as illustrated in Fig. 1. At first, the optimal selection of input features for obtaining the high correlated features with a manually specified range have been determined using Pearson correlation where the root mean square error and coefficient of determination are the lowest and highest. Then, by splitting the dataset into a training and a test model with a 70:30 ratio, significant features are extracted using the Boruta on RF classifier. The next phase involves using the Tree-based Pipeline Optimization Tool (TPOTClassifier) to assess the performance of 32 machine learning algorithms in order to select the best supervised model for this particular dataset. At the end, the web application with Streamlit [32] has been developed using the most optimize ML model to provide prediction outcomes with associated factors relating to mental illness.

### Dataset

To validate the effectiveness of the proposed approach, we examine our proposed algorithm on Young Minds Matter (YMM) dataset which is Australia's most recent nationally representative mental health survey of children and adolescents [33]. This survey has been conducted in collaboration between The University of Western Australia (UWA), Roy Morgan Research and the Australian Government Department of Health (AGDH) and collected data from a diverse, nationally representative survey of 4–17 years children and adolescents and their parents or caregivers. This data set has information on children’s mental disorders, child's learning, social conditioning, and healthy environment. The ethical approval of data collection process was obtained from the Human Research Ethics Committees of AGDH and UWA respectively [34, 35].

In YMM, a multi-stage, area-based random sample procedure has been conducted. It has been developed to represent Australian families with children and teenagers. If a family had more than one eligible child, a single child was randomly selected.

The survey included 6,310 parents/careers (55 percent of eligible households) of adolescents aged 4 to 17. The Diagnostic Interview Schedule for Children Version 4 (DISC-4) has been used in this dataset as a validated instrument for diagnosing mental health issues in children based on the Diagnostic and Statistical Manual of Mental Disorders Version 4 (DSM-4) criteria [12].

In the YMM dataset, the target class of indicating the presence of mental illness has significantly fewer observations of ‘yes’ status than the ‘no’ status. The ML algorithms prefer the larger class and, in some cases, ignore the smaller class where the minority class is the main interest. To overcome the methodological constraints of imbalance state of YMM dataset from the previous research [36], this study will deal with 1011 cases of participants aged from 4 to 17 years. From the parent reported data, these 1011 individuals have been selected based on the information that the child or adolescent went to doctor, mental health professional, psychiatrist or psychologist to get the confirmed mental health status.

### Data processing

The objective of this section is to explain about how the data has been processed to define the target variable and to identify the independent variables to make this analysis more effective.

#### Target variable

The variables influencing the state of OCD, SAD, and ADHD, as well as the status of the diagnosis continuation, have been chosen to measure the target variable. These are the questions/explanations of the variables that have been used to measure the target variable of OCD. For example, ‘Have you ever been told by a doctor or mental health professional that you have OCD?’, ‘Last year OCD duration at least two weeks’, ‘Last year OCD diagnosis for obsessions’, ‘Last month OCD diagnosis for obsessions’, ‘Last month OCD diagnosis for compulsions’, ‘Last year OCD diagnosis’, ‘Last month OCD diagnosis’, ‘Last year possible diagnosis for obsession’, ‘Last month possible diagnosis for obsession’.

Concerning SAD, inquiries include, ‘Have you ever been told by a doctor or mental health professional that you have any mental health problem?’,’ Last year SAD diagnosis (with criteria for disability)’,’ Last year SAD diagnosis’,’ Last month SAD diagnosis (with criteria for disability)’, ‘Last month SAD diagnosis’,’ Ever been told by a doctor or mental health professional that your child has separation anxiety?’.

Variables descriptions for measuring ADHD consist of ‘Have you ever been told by a doctor or mental health professional that you have ADHD?’,’ Last year ADHD diagnosis—any type (with impairment criterion)’,’ Last year ADHD Diagnosis- inattentive type (with impairment criterion)’,’ Last year ADHD diagnosis—hyperactive type (with impairment criterion)’, Last year ADHD diagnosis—any type—no age of onset (with impairment criterion),’ Last year ADHD diagnosis—combined type—no age of onset (with impairment criterion)’,’ Ever been told by a doctor or mental health professional that your child has ADHD?’.

The response of these variables has been confirmed by a doctor or mental health professional. If any of these variables has a true value, the target variable is validated as 1, otherwise it is 0.

#### Feature extraction

The purpose of this section is to minimise distraction from the primary target in each detection of mental illness by reducing data size due to the massive size of YMM data. All variables other than the target variables that are related to mental illnesses (like OCD, SAD, and ADHD) verified by a physician, professional mental health care provider, or any mental health service, as well as mental status, have been excluded from the training set as they have been considered for measuring the target variable. If a category contains more than 2000 ‘Unknown’ values, it has been eliminated. After removing these variables, there are 1041 categories left out of 2622 categorical variables for the feature extraction. Filtering method has been used to exclude 373 of these 1041 categories, which have data about ID, Cluster, house criteria, household income, region, country of birth for study child/primary carer/secondary carer, number of family members, their individual relation between the members living in the house, their main language to ensure the dataset used for this analysis is comprehensive, relevant, and accurate based on the fact that they do not have any impact or relationship with the target variable.

Once these variables are eliminated, the remaining variables are selected. The response of categories ‘Do not know’, ‘Refused’, ‘Missing’, ‘Not Available’, ‘null’ are replaced with ‘Unknown’ value. The values of the columns as ‘Yes – A lot’, ‘Yes – Minor’, ‘Yes – Minor difficulties’, ‘‘Yes – Severe difficulties’, ‘Yes – Sometimes’ are replaced with ‘Yes’. ‘Not at all’, ‘Never’ values are replaced with ‘No’. The column values have been encoded using factorize () to convert the string as numeric values.

To make analysis more efficient and minimize distraction from the primary target, after careful observation of data cleaning, 1474 categorical variables with ‘Yes’, ‘No’ values have been selected. Categorical data are replaced with 0 and 1 as the presence or the absence of the specific categorical data of these independent variables. In this study, due to the dichotomous nature (two categories of the variable) of these categorical variables, the Tetrachoric correlation of these independent factors has been performed in order to assess the strength of the association between two variables [37]. In fact, the tetrachoric correlation quantifies the degree of connection between two binary variables that have been artificially created.

As correlated variables, might lead to misleading feature importance, a range has been manually identified from the high correlated variables with the target variable. A set of correlated values between the given variables and the target variable has been chosen. The reason for choosing highly correlated variables with the target variable is that it minimises the dimensionality of a large set of dummy variables in YMM data whilst protecting significant information about the original data.

The best subset of input features has been picked at a stage where the Root Mean Square Error (RMSE) and coefficient of determination (R^2^) are the lowest and highest respectively. The optimal subset of features for OCD is 57 input features with RMSE and R^2^ of 0.24 and 0.27, respectively. 38 input features with RMSE and R^2^ of 0.30 and 0.46 respectively are selected as the best subset of features for ADHD. For SAD, the optimum subset is 64 input features where RMSE and R2 are 0.40 and 0.18 respectively.

### Methodology

The aim of this section is to evaluate which ML technique is the best suited to provide the improved outcomes for the obtained features in prediction model. The experiments have been carried out using Python 3.7.3 sci-kit-learn package to develop this strategy. In this experiment, firstly, the most significant input features have been determined using the Random Forest (RF) classifier with the Boruta method for unbiased and stable selection by partitioning the entire dataset into training and test datasets with high correlated features. In this research, there are several reasons to choose RF for feature selection. RF can handle classification with high accuracy and regression problems by capturing complex variable interactions as well as handling outliers and missing values. It comes with a feature importance metric, making it easy to select the most important features. Also, it can handle high-dimensional data, where the number of features is much larger than the number of samples. Overall, the combination of accuracy, interpretability, and scalability makes RF a popular choice for feature selection in this research.

In the experimental setting, firstly, the most significant input features have been determined using the Random Forest (RF) classifier with the Boruta method for unbiased and stable selection by partitioning the entire dataset into training and test datasets with high correlated features. The Tree-based Pipeline Optimization Tool (TPOTClassifier) can be a useful tool to automate the evaluation and optimization of multiple supervised ML algorithms, potentially saving time and effort in the model building process [38]. To develop a learning model incorporating the identified features, the performance of 32 ML supervised algorithms has been investigated using TPOTClassifier to determine which algorithm performs the best based on the chosen evaluation metric. According to the internal CV score of TPOTClassifier, the 3 best supervised learning algorithms out of 32 ML algorithms have been selected such as RF, Decision Tree (DT), and Gaussian Naive Bayes (GaussianNB) to generate the enhanced result of this predictive model. The model with the most optimal outcomes has been employed to develop the web application using an open-source Python framework Streamlit. The following subsections outline the operating principles of each ML algorithm.

### ML algorithms

A ML algorithm is a set of instructions that enables a computer program to learn from past experiences, detect patterns in data, and enhance its performance over time to generate predictions and make decisions without explicit programming. A supervised machine learning algorithm utilizes labelled data to train the model, in which the desired output is already established, and the algorithm employs this information to classify or predict new data. The supervised ML learning models analysed in this study are listed below.

#### Boruta algorithm

Boruta is a wrapper method on RF classifier, uses an all-relevant variable selection method, which takes into account all features that are significant to the outcome variable [39]. Here, predictor values are shuffled, joined with the original predictors, and then a random forest is built on the merged dataset. Then, to determine the significance of each variable, the original variables are compared to the random variables. The importance of each variable is measured by comparing it to the importance of the same variable when it is randomly shuffled with the other variables. If a variable has a higher importance score than the randomly shuffled version, it is considered significant and contributes to the predictive power of the model. However, if a variable has a lower importance score than the randomly shuffled version, it is not considered significant and is unlikely to contribute to the predictive power of the model. Finally, only variables that outperform the randomised variables are considered significant.

#### Tree-based pipeline optimization tool (TPOTclassifier)

The TPOTclassifier is a ML technique that uses genetic programming to find the best parameters and model ensembles. It comprises supervised classification models, preprocessors, selection methods, and any other science-related procedures to discover API-assessment estimators or transformers [40].

#### Random forest (RF)

The RF is an ensemble learning method that combines multiple decision trees to enhance the accuracy of predictions and reduce overfitting Each tree is trained on a random subset of the features, as opposed to all the features, in order to generate diverse decision trees. This process is known as feature bagging. The RF uses bagging by randomly selecting a subset of observations from the original dataset, constructing a decision tree based on majority ranking, and computing the average result. The number of trees and maximum tree depth are hyper-parameters of the RF method and show how many interactions in the model are assessed [41]. The importance of features is measured by the average over all trees. In this research, this approach has been used to rank the features based on their contribution to the model to select the features with higher importance for further analysis. The total number of trees is divided by the sum of the feature importance values on each tree with the following equation:

1 $$RFfi_{i} = \frac{{ \sum_{j \in\,all\,trees } normfi_{j} }}{T}$$

Here,

$RFfi_{i}$ = the feature importance, i calculated from all trees in the RF model.$normfi_{j}$ = the normalized feature importance for i in tree j

T = total number of trees.

#### Decision tree (DT)

Using tree data structure, decisions contribute as class labels, and leaf nodes serve as attributes on the decision trees. The test data or input pattern is represented by the nodes inside the tree. Based on the divide and conquer strategy, the internal nodes provide mutually exclusive and exhaustive findings for each test set [42, 43]. Using entropy, DT measures the information gain decide which features should be selected to reduce the uncertainty of the feature.

2 $$Information \,Gain = E\left( Y \right) - E(Y|X)$$

Here,

E(Y) = Entropy of the full dataset.

E(Y|X) = Entropy of the dataset given some feature.

#### Gaussian naïve bayes (GaussianNB)

The GaussainNB employs the Naive Bayes method and a Gaussian distribution without covariances to calculate the probability among feature values. In this, instances are allocated as class labels and the input feature values are represented as vectors [44]. The following formula is used to measure the probabilities for input values for each class through a frequency:

3 $$P\left( {X{|}Y = a} \right) = \frac{1}{{\sqrt {2\pi \sigma_{a}^{2} } }} e^{{\frac{{ - \left( {x - \mu_{a} } \right)^{2} }}{{2\sigma_{a}^{2} }}}}$$

Here,σ = variance of variable X computed for a given class a of Yμ = mean of the variable X computed for a given class a of Y

#### Streamlit

Streamlit, an open-source Python framework, has been used in this research to build a web application for the ML model. With the aid of relatively few lines of code, it is extremely capable of developing a variety of applications. There are several reasons why Streamlit has been selected for building this online application. First of all, as soon as the specified source file is saved, the code is automatically updated in the current kernel when one types in the source file. Moreover, with its cache method, datasets can be loaded in some expensive tasks. Most importantly, it is a relatively fast method for developing high-performance, reactive program in Python that makes use of a straightforward, declarative API [45]. Consequently, this has been used in this research.

### Performance measure

The performance of the proposed ML algorithms has been evaluated by accumulating True Positive (TP), True Negative (TN), False Positive (FP), and False Negative (FN) results by the confusion matrix. The accuracy, precision, sensitivity, specificity, AUC score in each ML model have been determined following the equations:

4 $${\text{Accuracy Rate}}\, = \,\frac{TP + TN}{{TP + FP + TN + FN}}$$

5 $${\text{Precision}}\, = \,\frac{TP}{{TP + FP}}$$

6 $${\text{Sensitivity}}\, = \,\frac{TP}{{TP + FN}}$$

7 $${\text{Specificity}}\, = \,\frac{TN}{{TN + FP}}$$

8 $${\text{F1 score}}\, = \,\frac{2\, \times \,Precision\, \times \,Recall}{{Precision + Recall}}$$

#### Area under receiver operating characteristic curve (AUC) score

The AUC score gives an indication of the overall performance of the classifier. It illustrates the True Positive Rate (sensitivity) versus the False Positive Rate ranges from 0 to 1 with higher values indicating better performance of the model at identifying positive cases [46].

#### K-fold cross validation (K-fold CV)

The K-Fold CV is widely used as accuracy estimator for reliability assurance of the method. There is no rigid law for deciding the value of K in the application of ML. The objective is to identify K with the minimal errors possible while maintaining the algorithm's capacity to properly anticipate outcomes when presented with new data. This is done repeatedly for various values of K [47]. According to the paper [48], an accurate estimation has a lesser bias when K = 10 (or 5).

## Results and discussions

In this section, we will first examine the representative features considered for this study. The proposed classification performance of the proposed models on the testing dataset is also covered in this section. This section concludes by demonstrating how the best fitted model is used with the most crucial input attributes to produce decision results for representative test situations.

### Exploration of significant features

In the YMM dataset, there are 73 cases of OCD, 235 cases of SAD, 168 cases of ADHD, however a majority class is regarded to have 938 ‘NonOCD’, 776 ‘NonSAD’ and 843 ‘NonADHD’ cases which encompasses 1011 cases of various mental disorder class. The random forest classifier has been applied with Boruta algorithm by partitioning dataset into training (70% observations) and test (30% observations) datasets, yielding 6,3 and 6 significant features for OCD [49], SAD and ADHD respectively among top ranked features shown in Table 2.

**Table 2:** Most significant features of obsessive–compulsive disorder (OCD) [41], separation anxiety disorder (SAD), and attention deficit hyperactivity disorder (ADHD) detection

#	YMM questionnaires Training set size: 707 Test set size: 304	Frequency proportion	
Most significant features for OCD identification		OCD (%)	NonOCD (%)
1	Not simply excessive worries about real-life problems	56.67	43.33
2	Attempts to ignore or suppress thoughts	37.84	62.16
3	Washing	100.00	0.00
4	Aimed at preventing or reducing stress	68.75	31.25
5	Checking: distress, time-consuming, or interferes with functioning	71.79	28.21
6	Counting: distress, time-consuming, or interferes with functioning	90.00	10.00
Most significant features for SAD identification		SAD (%)	NonSAD (%)
1	Wanted to stay at home and not go places without ATTACHMENT FIGURE	45.17	54.83
2	Reluctance/refusal to go to school/elsewhere because of fear of separation	62.84	37.16
3	Refuse to go to sleep away	50.99	49.01
4	Anxiety concerning separation from home/attachment figures	70.86	29.14
Most significant features for ADHD identification		ADHD (%)	NonADHD (%)
1	Senses a lack of close parental attention	62.18	37.82
2	Faces trouble paying attention	69.57	30.43
3	Leaves seat	63.69	36.31
4	Runs about or climbs excessively	64.46	35.54
5	Often on the go/driven by a motor	64.29	35.71
6	Behaves impulsive	86.24	13.76
7	Does not appear to be paying attention	68.45	31.55

### Experimental results

Table 3 provides overall classification performances for the proposed models in OCD [49], SAD and ADHD detection using RF, DT and GaussianNB in terms of accuracy, precision, specificity, sensitivity, f1 and AUC scores.

**Table 3:** Performance of proposed models of obsessive–compulsive disorder (OCD) [41], separation anxiety disorder (SAD), and attention deficit hyperactivity disorder (ADHD) detection

Diseases	Models	Accuracy	Precision	Specificity	Sensitivity	F1 Score	AUC
OCD	RF	0.92	0.57	0.97	0.36	0.44	0.7437
	DT	0.92	0.54	0.97	0.32	0.40	0.7037
	GaussianNB	0.91	0.76	0.96	0.50	0.60	0.7465
SAD	RF	0.83	0.73	0.95	0.42	0.53	0.7316
	DT	0.81	0.76	0.97	0.33	0.46	0.7010
	GaussianNB	0.79	0.62	0.91	0.46	0.53	0.7001
ADHD	RF	0.91	0.94	0.99	0.62	0.75	0.8256
	DT	0.87	0.76	0.96	0.50	0.60	0.7695
	GaussianNB	0.86	0.76	0.96	0.50	0.60	0.7695

**Fig. 2:**
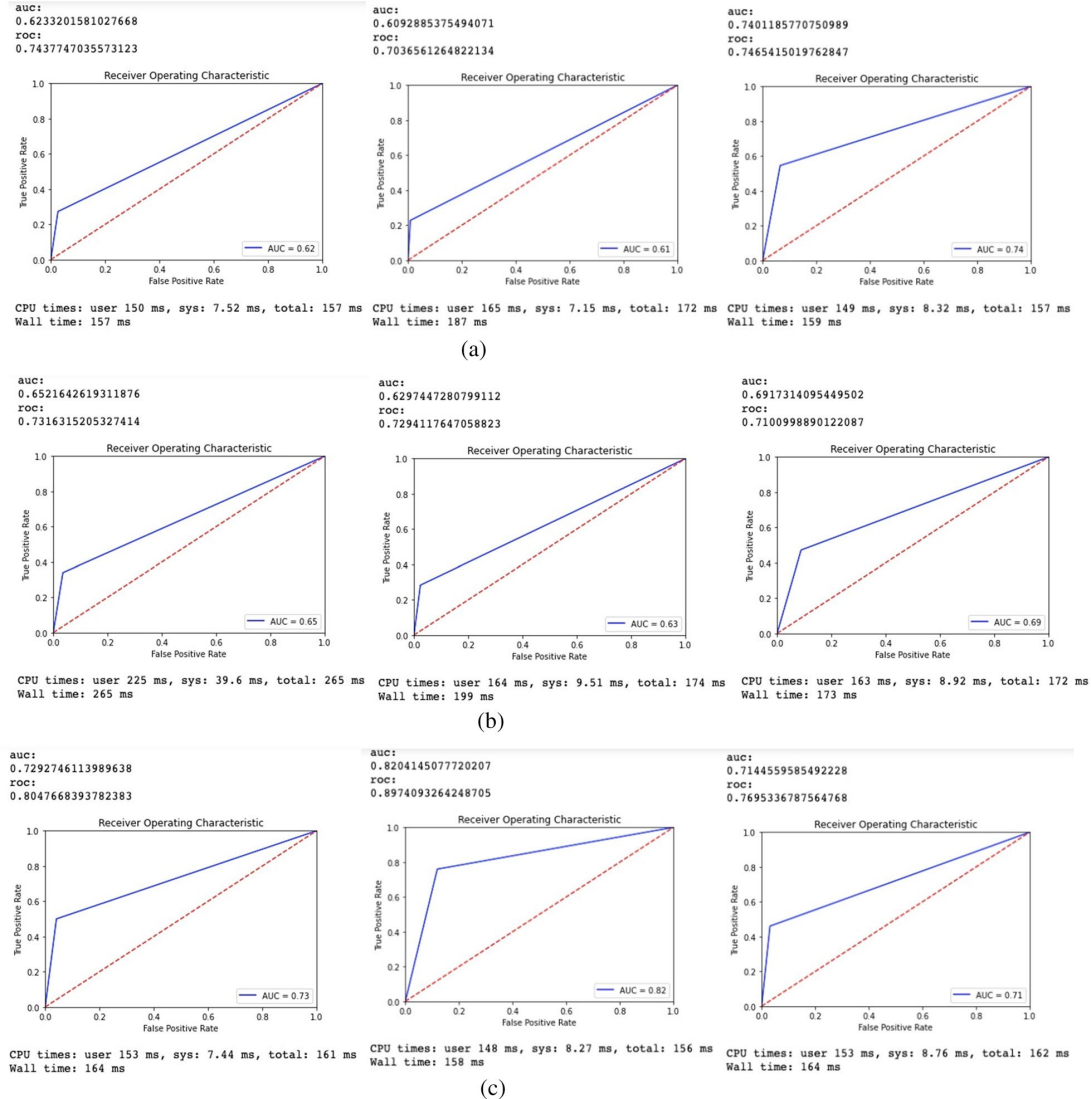
The ROC scores of RF, DT and GaussianNB in predicting **a** OCD [41], **b** SAD and **c** ADHD

From these performance metrics in Table 3, in terms of detecting OCD, the GaussianNB performs well in terms of precision (76%), sensitivity (50%), and F1 score (60%). GaussianNB has a specificity of 96%, which is somewhat lower than RF (97%). For detecting SAD, RF scored well in accuracy (83%), though DT performed well in terms of precision (76%) and specificity (97%); GaussianNB scored well in terms of sensitivity (46%). For SAD, RF and GaussianNB perform equally well (53%) in terms of F1 score. In terms of detecting ADHD, RF outperformed DT and GaussianNB. In addition, this study evaluates the AUC scores in Fig. 2.

The AUC values scored by the proposed models are within the acceptable range (0.70 to 0.80). These scores are slightly lower than the outstanding score of 0.80 [50]. In fact, the AUC score of RF is 0.83 in ADHD detection which is higher than the outstanding score (> 0.80). K-Fold cross validation scores have been shown in Table 4 to assess the detection model.

**Table 4:** Result of K-Fold cross validation of obsessive–compulsive disorder (OCD) [43], separation anxiety disorder (SAD), and attention deficit hyperactivity disorder (ADHD) detection

Diseases	Models	Threefold	Fivefold	Tenfold
OCD	RF	0.9335	0.9330	0.9352
	DT	0.9317	0.9267	0.9146
	GaussianNB	0.8262	0.8315	0.8244
SAD	RF	0.7593	0.7489	0.7847
	DT	0.7467	0.7478	0.7009
	GaussianNB	0.7676	0.7627	0.7579
ADHD	RF	0.8803	0.8860	0.8868
	DT	0.8209	0.8262	0.8262
	GaussianNB	0.8262	0.8315	0.8244

To assess the reliability of the methodologies, the accuracy of the results has been tested using the K-Fold cross validation accuracy estimator. Though RF scores the best in all terms of tenfold cross validation scores in Table 4 as well in terms of AUC scores in Table 3, sensitivity and f1 scores become important measures for determining the best model. Increased sensitivity indicates how well the predictive model can identify individuals who genuinely have the given conditions for a specific disease.

The YMM dataset shows, there are 73 cases of OCD, 235 cases of SAD, 168 cases of ADHD, however a majority class is regarded to have 938 ‘NonOCD’, 776 ‘NonSAD’ and 843 ‘NonADHD’ cases resulting in a completely imbalanced dataset containing 1011 cases. In such circumstances of classification imbalance, accuracy alone is insufficient, and the f1 score becomes an important criterion for selecting the optimal model. The f1 score is a harmonic mean of precision and recall, and its value increases as both precision and sensitivity increase. Therefore, in circumstances of classification imbalance, the model with the highest f1 score is the best model, even though its accuracy is lower [51].

Based on the findings in Table 3 for detecting SAD, GaussianNB and RF both scored well in terms of f1 score (53%), and DT scored the highest in terms of specificity (97%), however GaussianNB performed well in terms of sensitivity (46%) which is a significant indication for reliably identifying for positive cases (patients with disease). According to the results for ADHD detection in Table 3, RF outperformed than all other models in all terms (accuracy = 91%, precision = 94%, specificity = 99%, sensitivity = 62%, f1 score = 75%). All the algorithms applied in this study generated incredibly precise findings (specificity > 90%) for negative cases (patients without disease) as well.

### Discussion

After analysing the results, the developed algorithms in the framework are integrated into a web application using Streamlit. Table 5 shows how the decision has been determined using the Streamlit web application for 5 sample test cases with the best fitted model in ADHD, OCD and SAD prediction using the most significant input features.

**Table 5:** Attention deficit hyperactivity disorder (ADHD), obsessive–compulsive disorder (OCD) [43] and separation anxiety disorder (SAD) detection with test input data

Test samples for ADHD detection							
Lack of close parental attention	Trouble paying attention	Leaves seat	Runs about or climbs excessively	Often on the go	Behaves impulsive	Does not appear to be paying attention	Predicted ADHD
1	0	1	0	0	0	1	0
1	1	0	0	1	1	1	1
1	0	0	1	0	0	0	0
1	0	0	0	1	0	0	0
0	1	0	0	1	1	1	1

Test samples for OCD detection						
Not simply excessive worries about real-life problems	Attempts to ignore or suppress thoughts	Washing	Aimed at preventing or reducing stress	Checking: distress, time-consuming, or interferes with functioning	Counting: distress, time-consuming, or interferes with functioning	Predicted OCD
1	0	1	0	0	0	1
1	1	0	0	0	1	1
1	0	0	1	0	0	0
1	0	1	1	1	1	1
1	0	1	0	1	0	1

Test samples for SAD detection				
Wanted to stay at home and not go places without ATTACHMENT FIGURE	Reluctance/refusal to go to school/elsewhere because of fear of separation	Refuse to go to sleep away	Anxiety concerning separation from home/attachment figures	Predicted SAD
1	0	0	1	0
1	1	0	0	0
0	0	0	1	0
0	1	1	1	1
1	1	0	1	1

1 = Yes

0 = No

**Fig. 3:**
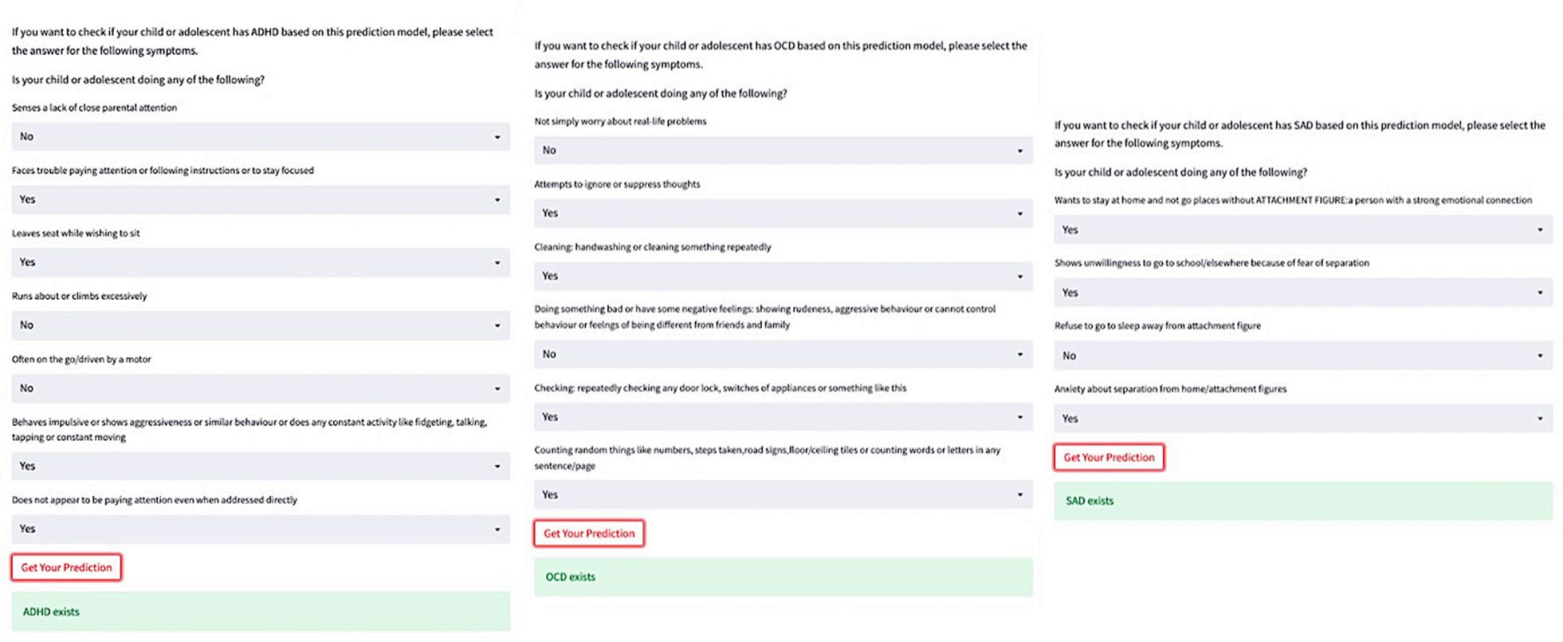
The interface for Streamlit web application in predicting ADHD, OCD and SAD

A screen shot of user interface for OCD, ADHD and SAD detection in Streamlit web app are shown in Fig. 3.

It is critical to comprehend the causes of mental diseases like OCD, SAD and ADHD in children and adolescents in order to effectively and quickly diagnose it for earlier detection and treatment. To identify significant patterns between input features with target domain in order to make adequate decisions, ML models need training data from a large dimensional dataset. In this study, YMM, Australia's most recent nationally representative mental health survey of children and adolescents has been used [34, 52].The top high correlated features have been identified from this large dimensional dataset. Moreover, this target variable in this dataset is also confirmed by a doctor, a mental health expert, and the status of diagnosis for OCD, SAD and ADHD.

The identified responsible factors have been clinically compared to the key symptoms of the identified mental disorders based on literature review, the ML model prediction assessment and dataset questionnaire evaluation. The six identified features of OCD have also been mentioned as important predictors in the previous research [53–55]. This study has revealed three significant features for SAD in the children and adolescents of Australia which have previously been defined as the major predictors in prior studies [56–58]. Furthermore, ADHD identification is difficult due to its proximity to other mental conditions with anxiety or mood disorder [59], this study has resulted six most significant features have also been recognised as major indicators of ADHD in previous studies [60–62]. These outcomes also indicate the efficacy of feature selection strategy in constructing the data template.

In the future, Convolutional neural network (CNN) can be used to manage high-dimensional inputs due to its features of appropriate input pre-processing and formatting [63]. In addition, various dimension reduction techniques, such as principal component analysis (PCA) and independent component analysis (ICA), can be used to compare the number of features by identifying the most important features in order to reduce the load on the classifier by simplifying the input data [64]. Our future works will include incorporating CNNs along with these dimension reduction techniques for efficient handling of high-dimensional inputs to further optimise the data modelling approach.

This study validates a new framework to provide the best fitting model for OCD, SAD, and ADHD identification with the most important indications of children and adolescents in the Australian context using Australia's most recent nationally representative mental health survey of children and adolescents. In Australia, where the ratio of psychiatrists to the population is 14,000:100,000, it is extremely challenging to obtain an appointment with a certified psychiatrist for mental health diagnoses. When parents, caregivers, or school authorities are uncertain if they need to consult a medical specialist, this validated framework will be highly useful for them to discover these mental diseases early on a primary basis to start the treatment as early as possible.

### Limitation of the study

There are some limitations to this study that should be addressed. Specifically, the sample of the study is restricted to Australia, and the efficacy of the algorithms may vary across populations. The results may not be representative of the entire population as the survey was conducted only in Australia. Due to the yes-or-no classification methodology used in this study, the severity of these mental disorders cannot be determined. Another limitation of this research is the exclusion of 'Unknown' categories, which can result in the loss of valuable information and potentially skew the findings and conclusions. However, the outcomes of this model clearly demonstrate the effectiveness of data building template. In the near future, the application of this method will be extended to another clinical huge data to examine the performance of the model and detection will be analysed by measuring the severity level.

## Conclusion

In this paper, a framework has been validated to detect the mental illnesses such as ADHD, OCD and SAD at their early stage using the ML models. A web application has been designed with these ML models to present the actual signs of these mental illnesses that children and adolescents are encountering with a primary focus in the Australian context. Amidst significant variation in model performance, GaussianNB has been discovered to be an effective and accurate classifier for diagnosing OCD and SAD; RF outperforms than all other classifier for detecting ADHD using YMM, a large dimensional dataset on the mental health of children and adolescents in Australia. All three algorithms (RF, DT, and GaussianNB) accomplished great performance in terms of confusion matrix parameters, K-fold cross validation results and AUC score with the minimum number of features incorporated. These performances have shown the capabilities of TPOTClassifier in the model.

The results also show how the YMM dataset has a substantial predictive impact on OCD, ADHD and SAD. There are several existing predictive models for these mental illnesses’ detection; however, this model is more accurate and instructive in predicting child and adolescent OCD, ADHD and SAD in the Australian context due to its large dimensional dataset, optimal feature set, and most importantly, high precision, specificity, and F1 score in prediction. Moreover, this model is integrated into a web application using Streamlit to make the model as a viable tool to build and function. This model can therefore be used as the basis for identifying OCD, ADHD, and SAD in children and adolescents. The parents, caregivers or teachers whoever are not sure whether their child, adolescent or student might have suffered from these mental illnesses, they can use this online application. To avoid long-term difficulties, this approach can assist them recognise the disease early and initiate treatment as soon as possible.

## Data Availability

The code is included in the files labelled “Supplemental Material”.
